# Pancreatogastric fistula in chronic pancreatitis: a rare and life-threatening cause of recurrent massive upper gastrointestinal bleeding

**DOI:** 10.1093/jscr/rjaf619

**Published:** 2025-09-06

**Authors:** Sanduni Wijerathne, Gayathri Bandaranayake, Anjana Abeysinghe, Sivasuriya Sivaganesh, Nilesh Fernandopulle, Duminda Subasinghe

**Affiliations:** Department of Surgery, Faculty of Medicine, University of Colombo, No. 25, Kynsey Road, Colombo 8, Sri Lanka; Department of Surgery, Faculty of Medicine, University of Colombo, No. 25, Kynsey Road, Colombo 8, Sri Lanka; University Surgical Unit, The National Hospital of Sri Lanka, Colombo 10, Sri Lanka; Department of Surgery, Faculty of Medicine, University of Colombo, No. 25, Kynsey Road, Colombo 8, Sri Lanka; University Surgical Unit, The National Hospital of Sri Lanka, Colombo 10, Sri Lanka; Department of Surgery, Faculty of Medicine, University of Colombo, No. 25, Kynsey Road, Colombo 8, Sri Lanka; University Surgical Unit, The National Hospital of Sri Lanka, Colombo 10, Sri Lanka; Department of Surgery, Faculty of Medicine, University of Colombo, No. 25, Kynsey Road, Colombo 8, Sri Lanka; University Surgical Unit, The National Hospital of Sri Lanka, Colombo 10, Sri Lanka

**Keywords:** pancreatogastric fistula, chronic pancreatitis, massive upper gastrointestinal bleeding

## Abstract

Pancreatogastric fistulas are rare but serious complications of chronic pancreatitis that can lead to life-threatening gastrointestinal bleeding due to erosion of nearby blood vessels. We present a case of a 43-year-old man with chronic calcific pancreatitis and a history of alcohol misuse, who experienced recurrent hematemesis and melena over 2 months. Despite multiple endoscopies and transfusions, the bleeding source remained unidentified until imaging revealed a fistulous tract between the pancreas and the posterior gastric wall. After failed endoscopic control, surgery confirmed the fistula and bleeding vessel, which were managed with vessel ligation and gastric resection. This case underscores the need to consider pancreatogastric fistulas in patients with chronic pancreatitis and unexplained gastrointestinal bleeding, particularly when standard investigations are inconclusive and conservative measures fail.

## Introduction

Pancreatogastric fistulas are abnormal communications between the pancreas and the stomach, which can arise from both benign and malignant conditions. While pancreatogastric fistulas have been documented in both acute and chronic pancreatitis, they are notably rare in chronic cases. Benign fistulas typically develop due to pancreatic pseudocysts, necrosis, or ductal hypertension secondary to chronic inflammation [1, 2]. Malignant causes include pancreatic adenocarcinoma and intraductal papillary mucinous neoplasms (IPMNs), where tumor invasion leads to fistulation [3]. In contrast to acute pancreatitis, where fistulas commonly result from spontaneous pseudocyst rupture into the stomach, chronic pancreatitis rarely leads to fistula formation due to fibrosis and scarring. When present, pancreatogastric fistulas in chronic pancreatitis can cause life-threatening complications, such as massive upper gastrointestinal bleeding from erosion into surrounding blood vessels [4, 5]. This report presents a rare case of pancreatogastric fistula in chronic pancreatitis, manifesting as recurrent massive upper gastrointestinal bleeding, which required surgical intervention. The case underscores the diagnostic challenges and highlights the importance of considering pancreatogastric fistulas as a rare but potentially fatal complication of chronic pancreatitis.

## Case presentation

A 43-year-old male with a history of alcohol misuse, who had not undergone prior medical evaluations, presented with recurrent hematemesis and melena over the past 2 months. His hemoglobin was 5.9 g/dl. Prior to admission, he had required multiple blood transfusions and underwent several upper gastrointestinal endoscopies that failed to identify the source of bleeding. Retrospective assessment revealed a history of recurrent upper abdominal pain suggestive of pancreatitis, although a formal diagnosis had not been established.

Upon admission, the patient was resuscitated with three units of blood. Upper gastrointestinal endoscopy revealed a visible bleeding vessel on the mid-body of the posterior gastric wall. Endoscopic ultrasound (EUS) suggested the vessel likely originated from the splenic vein adjacent to the bleeding site. A CT mesenteric angiogram showed no active bleeding. However, a CT scan demonstrated chronic calcific pancreatitis involving the distal pancreas, with a fistulous tract extending from the pancreas to the posterior stomach wall. No splenic artery pseudoaneurysm was noted.

**Figure 1:**
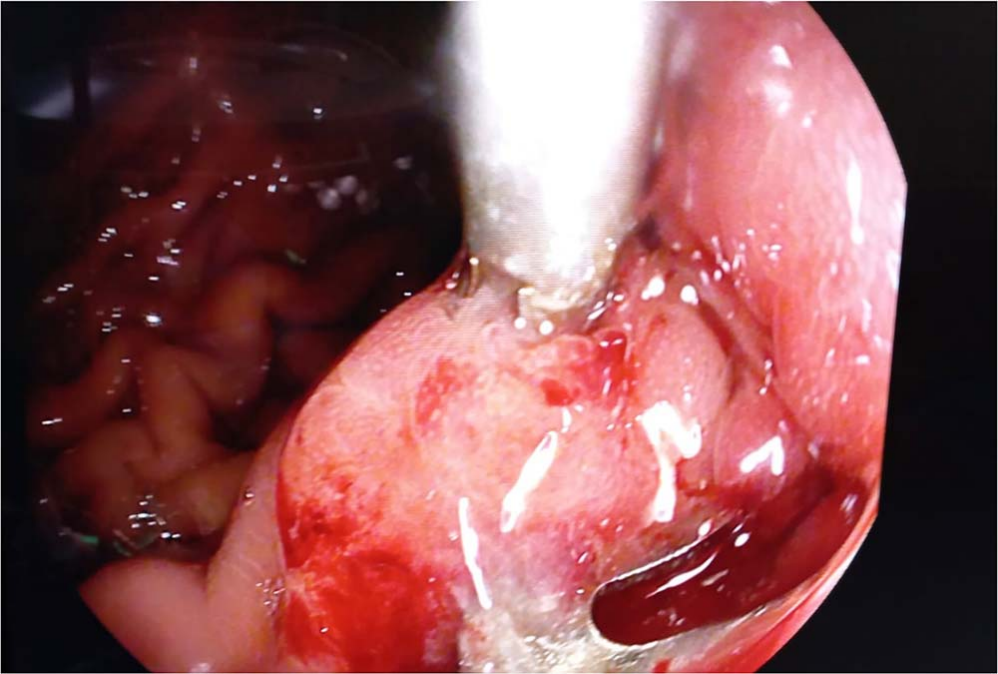
Intraoperative endoscopic view of pancreaticogastric fistula with previous endoscopic clip.

Endoscopic clipping of the bleeding vessel was attempted but failed to control the hemorrhage, and the patient continued to experience hematemesis (Fig. 1).

**Figure 2:**
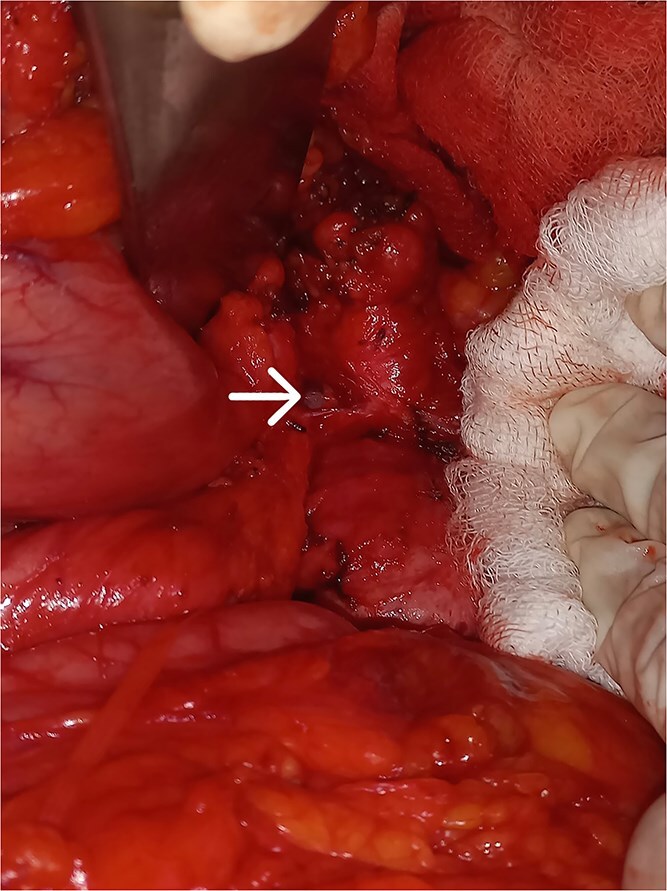
Pancreaticogastric fistula.

**Figure 3:**
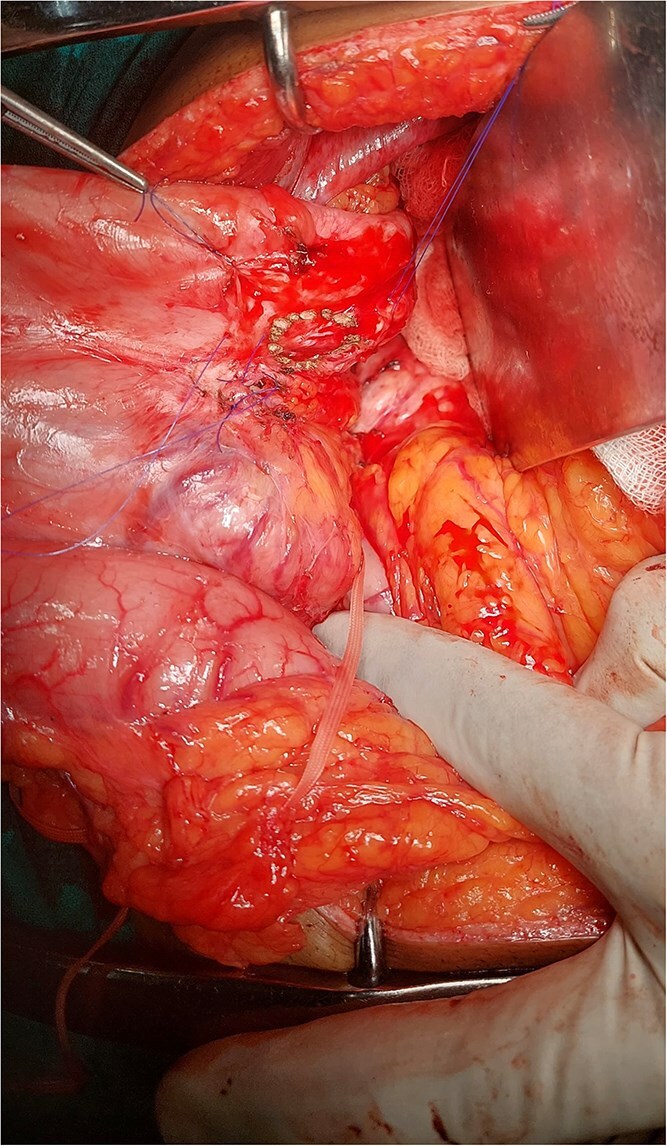
Stomach posterior wall cuff.

**Figure 4:**
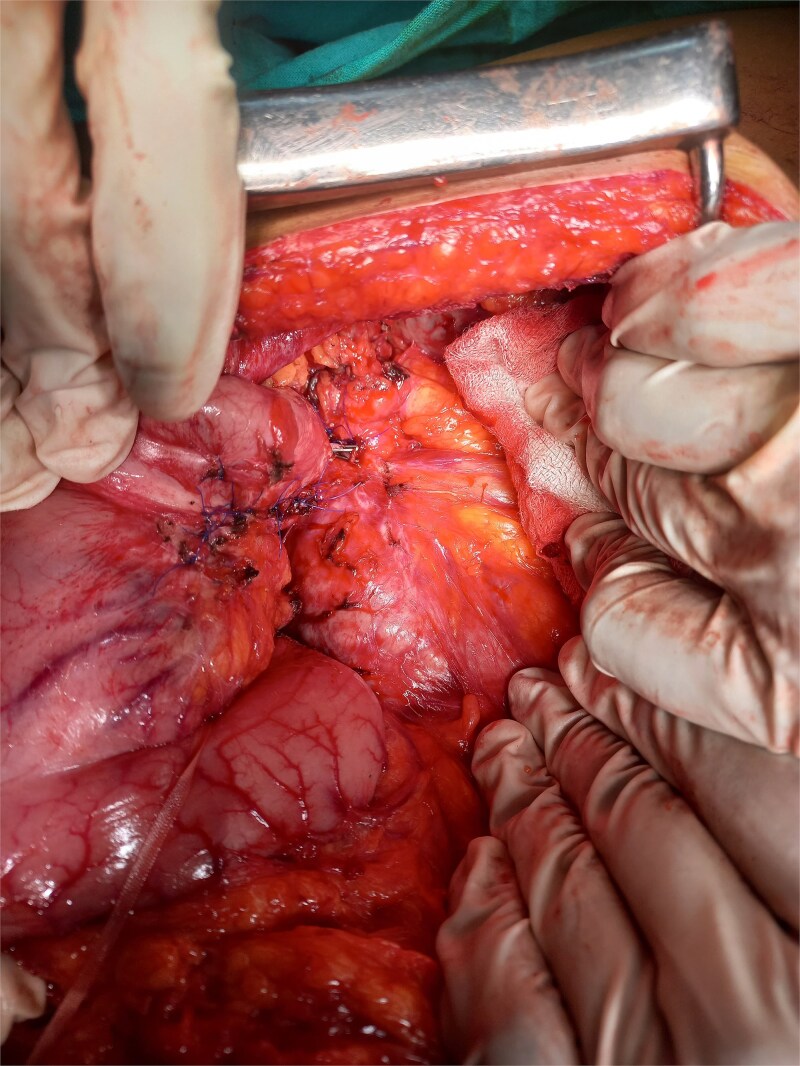
Gastric wall repair.

Given the persistent, life-threatening bleeding, urgent laparotomy was performed. Intraoperative findings revealed a hardened distal pancreas, densely adherent to the posterior stomach wall, with evidence of chronic pancreatitis. A fistulous tract was identified near the greater curvature of the stomach (Fig. 2), and an arterial bleeder was confirmed via intraoperative endoscopy. The bleeding vessel was ligated, and a small portion of the stomach, including the fistulous tract, was resected. The gastric defect was closed with 3-0 polypropylene sutures and reinforced with an omental patch (Figs 3 and 4).

## Discussion

Pancreatic inflammation can lead to both internal and external fistulas. While external fistulas are typically a result of surgery, internal fistulas can arise spontaneously in the setting of pancreatitis [1]. These internal fistulas can involve adjacent bowel loops, the stomach, oreven the mediastinum, potentially resulting in massive hematemesis or sepsis [6]. Our patient presented with recurrent hematemesis, a well-documented feature in pancreatogastric fistulas, which occur in approximately 54% of cases. However, pancreatogastric fistulas are the rarest form of pancreatoenteric fistulas and represent an exceptionally uncommon cause of upper gastrointestinal bleeding requiring surgical intervention. While internal fistulation is commonly seen in malignancies such as intraductal papillary mucinous neoplasms (IPMNs), it can also occur in pancreatitis due to pseudocysts, hematomas, or abscess drainage [4, 6]. Although pancreatic ductal dilatation is often present, it was not observed in our patient. Imaging failed to reveal a cyst or necrotic collection, which suggests that the fistula may have developed through mechanisms other than spontaneous rupture of a pseudocyst. This case represents the fourth reported instance of a pancreatogastric fistula in chronic pancreatitis, underscoring its rarity as a cause of massive upper gastrointestinal bleeding. Preoperative diagnosis of pancreatogastric fistulas can be challenging, often requiring intraoperative identification [3]. Despite multiple upper gastrointestinal endoscopies, EUS, and CT scans, the diagnosis in our case was confirmed during surgery. One difficulty in diagnosing pancreatogastric fistulas is the challenge of locating the fistula opening during endoscopy. In cases where there is communication with the main pancreatic duct, ERCP-guided dye injection may assist in identifying the tract [4, 6]. However, few chronic pancreatitis-associated cysts communicate with the pancreatic duct, which contributes to the underreporting of pancreatogastric fistulas in such patients [5]. Conservative management of pancreatic fistulas typically includes nasogastric suction, electrolyte replacement, and nutritional support, with spontaneous healing being possible insome cases [1]. Some authors suggest that fistulas may reduce the frequency of pancreatitis episodes by facilitating drainage and relieving ductal pressure. In certain situations, pancreatogastric fistulas associated with abscesses may resolve spontaneously, leading to improved outcomes [7]. However, early surgical intervention is recommended for colonic fistulas, which have a poorer prognosis [6]. In cases of severe hemorrhage, as observed in our patient, surgical intervention remains the only life-saving option when endoscopic measures fail [3, 6].

## Conclusion

Pancreatogastric fistulas are a rare but potentially life-threatening complication of chronic pancreatitis, particularly as a cause of massive upper gastrointestinal bleeding. Given their rarity and diagnostic challenges, a high index of suspicion is necessary. This case highlights the importance of considering pancreatogastric fistulas as a rare but serious etiology of gastrointestinal hemorrhage that necessitates definitive surgical treatment.
